# Alleviation of Intestinal Inflammation by Oral Supplementation With 2-Fucosyllactose in Mice

**DOI:** 10.3389/fmicb.2019.01385

**Published:** 2019-06-19

**Authors:** Thomas Grabinger, Jesus Francisco Glaus Garzon, Martin Hausmann, Annelies Geirnaert, Christophe Lacroix, Thierry Hennet

**Affiliations:** ^1^Institute of Physiology, University of Zurich, Zurich, Switzerland; ^2^Department of Gastroenterology and Hepatology, University Hospital Zurich – University of Zurich, Zurich, Switzerland; ^3^Laboratory of Food Biotechnology, Department of Health Sciences and Technology, ETH Zurich, Zurich, Switzerland

**Keywords:** colitis, IBD, milk oligosaccharide, fucose, carbohydrates, microbiota, *Lachnospiraceae*, *Ruminococcus*

## Abstract

Milk oligosaccharides exert a prebiotic action that contributes to the development of the infant gut microbiota during lactation. Given that milk oligosaccharides remain intact after passage through stomach and small intestine, they can potentially influence the composition of the gut microbiota when ingested as dietary supplements after weaning. To address the regulatory effects of specific oligosaccharides in colitis linked to the microbiota composition, we have supplemented interleukin-10 null (*Il10*^-/-^) mice with four fucosylated and sialylated oligosaccharides. We found that oral supplementation with 2-fucosyllactose significantly decreased the severity of colitis as displayed by reduced inflammatory marker expression, histological and diarrhea scores, an increased epithelial integrity and less pronounced colon shortening. Oral supplementation with 2-fucosyllactose led to a marked expansion of the commensal *Ruminococcus gnavus*, which was accompanied by an enhanced cecal concentration of propionate. Decreased activation of immune cells by *R. gnavus* was confirmed by reconstitution of antibiotic-treated *Il10*^-/-^ mice and by stimulation of dendritic cells *in vitro*. This study demonstrates that post-weaning administration of specific oligosaccharides can shift the composition of the gut microbiota to lessen chronic inflammation as observed in *Il10*^-/-^ mice. The expansion of *R. gnavus* sets a positive microbial environment at the cost of pro-inflammatory Gram-negative bacteria, thereby lowering intestinal inflammation.

## Introduction

The gastrointestinal tract is colonized by a complex ecosystem of microorganisms that are referred to as the intestinal microbiota (Lynch and Pedersen, 2016). It consists of trillions of microbes, mainly found in the colon, from hundreds of different species that live in a mutually beneficial interdependency with the host organism (Eckburg et al., 2005; Xu et al., 2007). Playing an important role in immune homeostasis, vitamin provision as well as nutrient utilization of indigestible components, the intestinal microbiota fulfills critical physiological roles within the colonized host (Mazmanian et al., 2005; LeBlanc et al., 2013; Ramakrishna, 2013; Honda and Littman, 2016). In the gut, energy for bacterial growth can be derived through processes such as fermentation of dietary and host carbohydrates (Chassard and Lacroix, 2013). Bacterial fermentation products, such as the short chain fatty acids (SCFA) propionate, acetate and especially butyrate have beneficial effects on host glucose homeostasis, protect from inflammation and act as important energy sources for colonocytes (Sun et al., 2017). Furthermore, an established intact microbial community also protects the host against pathogen colonization and expansion by competing for nutrients and space (Gill et al., 2011). In contrast to an intact microbiota, a destabilized microbial community is referred to as dysbiosis (Weiss and Hennet, 2017). Several pathological conditions such as obesity, diabetes and inflammatory bowel disease (IBD) correlate with dysbiosis (Turnbaugh et al., 2006; Maloy and Powrie, 2011; Winer et al., 2017). For instance, a reduced microbial diversity featuring reduced Firmicutes and outgrown Proteobacteria are typical hallmarks of dysbiosis in IBD (Swidsinski et al., 2002). Beside other factors including antibiotic treatment and bacterial toxins, the host diet contributes to microbial shifts, especially in the initial postnatal colonization of the gut (Hussey et al., 2011; Zeissig and Blumberg, 2014; Backhed et al., 2015). Accordingly, nutrients provided by breast milk, usually the primary diet in the first period of life, are critical for defining a healthy microbiota by supporting growth of beneficial bacterial strains (Pannaraj et al., 2017). Consequently, among the many beneficial effects that breastfeeding provides for infants, one is to lower the occurrence of Crohn’s disease or ulcerative colitis later in life (Xu et al., 2017). Representing a prominent component of breast milk, oligosaccharides are of particular interest in this regard (Hennet and Borsig, 2016).

In human breast milk, oligosaccharides represent a structurally diverse group of about 200 soluble carbohydrates that sum up to 5–10 g per liter (Zivkovic et al., 2011). The composition of milk oligosaccharides varies between individual mothers and over the course of lactation (Coppa et al., 1993; Marx et al., 2014). To date, the functional significance for this oligosaccharide diversity is still debated. In sharp contrast to the essential contribution of breast milk as a source of energy for the infant, milk oligosaccharides are indigestible to the newborn (Sela and Mills, 2010). In fact, mammals lack the enzymatic machinery necessary to release monosaccharides, such as fucose, N-acetylglucosamine and sialic acid, which are common building blocks of milk oligosaccharides (Engfer et al., 2000). Instead, milk oligosaccharides act as prebiotics, used as nutrients by selected bacterial groups, such as bifidobacteria (Davis et al., 2016). In addition to their prebiotic effect, milk oligosaccharides also affect host-microbe interactions, for example by acting as receptor decoys preventing pathogen binding to host epithelial cells (Newburg et al., 2005). Moreover, milk oligosaccharides affect gene expression in intestinal epithelial cells, influencing their differentiation, survival and surface glycan expression (Angeloni et al., 2005; Kuntz et al., 2008).

Previous studies have shown that prebiotic polysaccharides used as food additives, such as galacto-oligosaccharides or fructo-oligosaccharides of the inulin type, have a beneficial effect in Crohn’s disease and ulcerative colitis patients (Wilson and Whelan, 2017). The mechanisms underlying the positive effect of prebiotic carbohydrates on colitis and the role of the microbiota in these processes are largely unknown. Given the beneficial impact of milk oligosaccharides in newborns, these oligosaccharides may also exert positive effects on dysbiotic microbiota after lactation (Peterson et al., 2013). To date, little is known about the impact of milk oligosaccharides supplemented in children post-weaning and in adults (Elison et al., 2016). To clarify the role of naturally occurring oligosaccharides featuring fucosyl- and sialyl-residues on the course of colitis, we have investigated the effects of the orally supplemented oligosaccharides 2-fucosyllactose (2FL), 3-fucosyllactose (3FL), 3-sialyllactose (3SL), and 6-sialyllactose (6SL) on the course of intestinal inflammation in interleukin-10 null (*Il10*^-/-^) mice after weaning. These trisaccharides represent the simplest fucosylated and sialylated carbohydrates that remain uncleaved by host-encoded carbohydrate hydrolases.

## Results

### 2FL Supplementation Decreased Inflammation in *Il10*^-/-^ Mice

The lack of the immunosuppressive effects mediated by interleukin-10 leads to a progressive enterocolitis related to the continuous stimulation of the mucosal immune system by the microbiota (Kuhn et al., 1993). To assess the impact of naturally occurring prebiotic oligosaccharides on the course and severity of colitis, weaned wildtype (WT) and *Il10*^-/-^ mice were supplemented with oligosaccharides in drinking water starting at 3 weeks of age for 4 weeks. A concentration of 5 mM of either lactose (Lac), 2FL, 3FL, 3SL, or 6SL was provided with the drinking water. This concentration was chosen, because it fails to induce any signs of discomfort such as soft stool, diarrhea, decreased weight gain and behavioral changes. Mice may display intolerance symptoms related to high lactose concentrations after down-regulation of lactase expression post-weaning (Fang et al., 2006).

**FIGURE 1:**
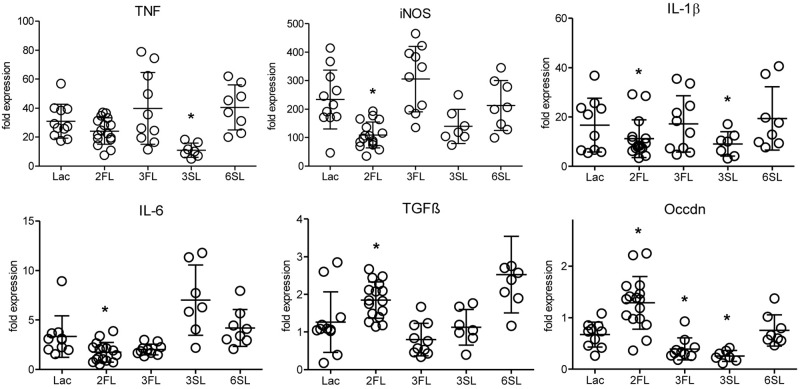
Influence of oligosaccharide supplementation on expression of inflammation markers in *Il10*^-/-^ mice. *Il10*^-/-^ mice (open circles) were supplemented with oligosaccharides for 4 weeks. Expression levels of inflammation markers were assessed by qPCR of distal colonic tissue at 7 weeks of age. Expression of the iNOS, the cytokines IL-1β, IL-6, TGFβ, TNF, and the tight junction protein occludin was determined. β-actin was used for normalization and the sample that represents median from Lac supplemented WT mice was used as reference sample according to the ΔΔC_t_ method. Each group consisted of at least six animals (*N* = 6–12) from 2 (3SL, 6SL), 3 (3FL), or 4 (Lac, 2FL) independent supplementation experiments. Error bars indicate SD. *P*-values were determined by one-way ANOVA with Dunnett Post-test and are indicated by asterisk (^∗^) if significantly different from control (Lac) condition (*p* < 0.05).

The analysis of inflammatory markers, cytokines, and markers of epithelial integrity in the distal colon of mice after 4 week of oligosaccharide supplementation revealed that one oligosaccharide, 2FL, led to significantly decreased expression of the pro-inflammatory markers iNOS, IL-1β and IL-6 (Figure 1). At the same time, the expression of TGFβ, a factor involved in wound healing-associated tissue remodeling, as well as occludin, a tight junction protein associated with epithelial integrity, were increased in *Il10*^-/-^ mice after 2FL supplementation. By contrast, the structurally related oligosaccharide 3FL and the sialylated oligosaccharide 6SL did not induce any changes in *Il10*^-/-^ mice after oral supplementation. The other sialylated oligosaccharide, 3SL, decreased IL-1β and TNF expression, while it also decreased expression of occludin (Figure 1). The same trend was observed in proximal colon tissue of *Il10*^-/-^ mice, which showed a lower degree of inflammation when compared to distal colon (Supplementary Figure S1A). None of the oligosaccharides tested changed the expression of inflammation markers in WT colonic tissue (Supplementary Figure S1B). These data indicated that specific oligosaccharides influenced the course of intestinal inflammation. Interestingly, the structurally related fucosylated oligosaccharides 2FL and 3FL exerted different effects on the expression of marker genes, thus underlining the importance of the glycosidic linkages for the biological roles of oligosaccharides.

**FIGURE 2:**
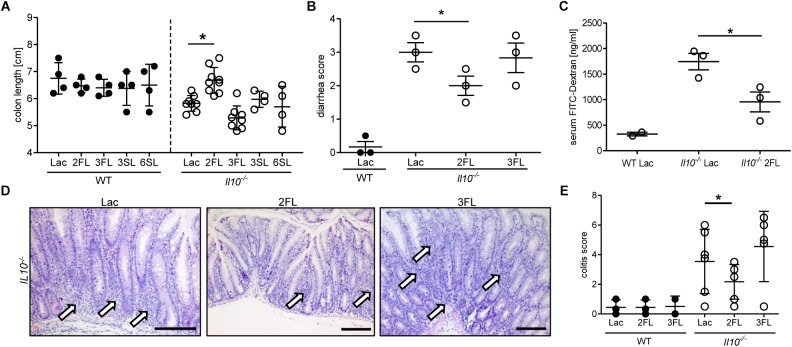
Signs of decreased colonic inflammation after 2FL supplementation. WT (filled circles) and *Il10*^-/-^ (open circles) were supplemented with Lac, 2FL, or 3FL for 4 weeks. **(A)** Colon length and **(B)** diarrhea scores (Sakai et al., 2013). **(C)** Intestinal permeability of supplemented *Il10*^-/-^ and WT mice as determined by serum FITC-dextran levels after gavage. **(D)** Representative images of H&E stained colonic tissue sections. Massive leukocyte infiltration sites are indicated by white arrows. Scale bar = 100 μm. **(E)** Colitis score of colonic tissue sections. Each group consisted of at least three animals (*N* = 3–7) from 2 independent supplementation experiments. Error bars indicate SD, *P*-values were determined by one-way ANOVA with Dunnett Post-test, and indicated by asterisk (^∗^) if significant (*p* > 0.05).

The anti-inflammatory effect of 2FL supplementation on colitis in *Il10*^-/-^ mice was confirmed by measuring the changes in colon length in oligosaccharide-supplemented WT and *Il10*^-/-^ mice. Only 2FL supplementation maintained a normal colon length in *Il10*^-/-^ mice, whereas the other oligosaccharides had no effect on the shortening of the colon occurring through inflammation (Figure 2A). Similarly, supplementation with 2FL decreased diarrhea (Figure 2B) and intestinal permeability (Figure 2C), in line with the observed increased expression of occludin in 2FL supplemented *Il10*^-/-^ mice (Figure 1). Mucosal damage was examined histologically and scored in distal colon sections of Lac, 2FL, and 3FL supplemented *Il10*^-/-^ mice. Whereas Lac and 3FL supplemented animals showed leukocyte infiltration, lamina propria swelling, cryptitis, goblet cell loss and epithelial damage, these traits were markedly decreased in 2FL supplemented mice (Figure 2D). Blind scoring of histological sections confirmed that supplementation with 2FL, but not 3FL reduced colitis in comparison to control treatment with Lac (Figure 2E).

### Oligosaccharide Supplementation Affected the Intestinal Microbiota in *Il10*^-/-^ Mice

**FIGURE 3:**
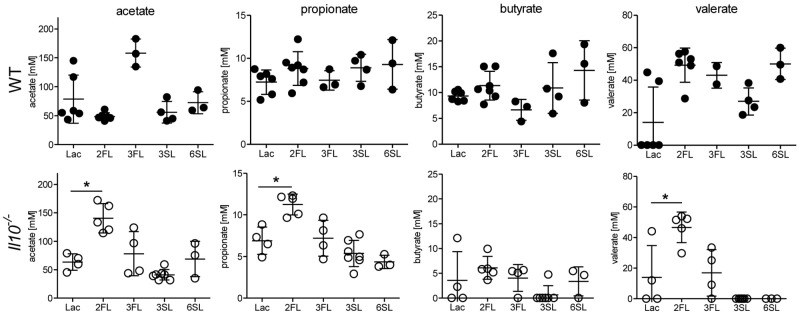
Oligosaccharide supplementation change short chain fatty acid concentration in cecal fluid. Concentrations of the SCFAs acetate, propionate, butyrate, and valerate were analyzed in cecal fluids of WT mice (upper row) and *Il10*^-/-^ mice (lower row) after 4 weeks of oligosaccharide supplementation in drinking water. Each group consisted of at least three animals (*N* = 3–7) from 2 (1 for 3FL and 6SL) independent supplementation experiments. Error bars indicate SD, *P*-values were determined by one-way ANOVA with Dunnett Post-test and are indicated by asterisk (^∗^) if significant (*p* > 0.05).

Considering the selective effects of 2FL against related oligosaccharides, we anticipated a similar variable impact of each oligosaccharide supplemented on the intestinal microbiota. Shifts in intestinal microbial community are likely to influence the local concentrations of bacterial fermentation products, such as SCFAs (Macfarlane and Macfarlane, 2003). Accordingly, we measured the cecal levels of SCFAs in oligosaccharide-supplemented WT and *Il10*^-/-^ mice. Whereas most of the oligosaccharides did not change SCFA levels, 2FL supplementation in *Il10*^-/-^ mice was paralleled by increased cecal concentrations of acetate, propionate, and valerate (Figure 3). To further document the shifts in intestinal microbial composition over the course of 2FL and 3FL supplementation, fecal pellets were collected on day 0, day 14, and day 28 of supplementation, and bacterial DNA diversity was assessed by 16S rRNA sequencing. Oligosaccharide supplementation did not appear to alter microbial species richness in neither WT nor *Il10*^-/-^, as shown by alpha diversity rarefaction curves (Supplementary Figure S2A). Microbial diversity was, however, lower in *Il10*^-/-^ mice when compared to WT animals, which is in accordance to previous findings (Gong et al., 2016). Taxonomic evaluation of sequencing data at the family level revealed considerable differences in WT vs. *Il10*^-/-^ mice (Supplementary Figure S2B), with a higher abundance of *Porphyromonadaceae* and a lower abundance of *Bacteroidaceae* and *Lachnospiraceae* in WT animals in comparison to *Il10*^-/-^ mice (Supplementary Figure S2C). Notably, some operational taxonomic units (OTU) that were abundant in WT mice appeared to be almost completely absent in *Il10*^-/-^ mice, such as *S24-7*, *Prevotellaceae*, and *Desulfovibrionaceae*.

**FIGURE 4:**
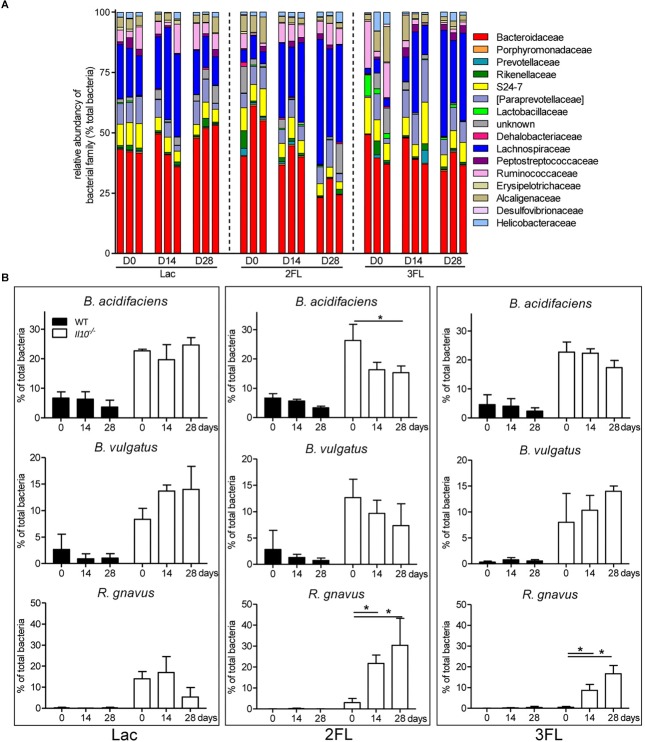
Shift in microbiota at family level after 2FL supplementation in IL-10^-/-^ mice. Bacterial DNA was isolated from fecal pellets of 2FL, 3FL and Lac control supplemented mice on day 0 (D0), day 14 (D14), and day 28 (D28) in *Il10*^-/-^ mice. Bacterial composition of each sample was assessed by 16S rRNA sequencing. Individual biological samples of one representative supplementation experiment (*N* = 3) are shown. Each taxa is shown as the percentage of total OTUs within the sample. Only the bacterial taxa representing at least 1% of total identified sequences are presented. **(A)** Bacterial families are shown as percentage of total OTUs. Each individual replicate is shown. **(B)** Effect of oligosaccharide supplementation on relative levels of *B. acidifaciens*, *B. vulgatus*, and *R. gnavus*. Mean values are shown, error bars indicate SD. *P*-values were determined by one-way ANOVA with Dunnett Post-test and are indicated by asterisk (^∗^) if significant (*p* > 0.05).

Oral supplementation of *Il10*^-/-^ mice with Lac did not affect the microbial composition significantly. By contrast, the ingestion of 2FL and 3FL induced a marked increase in *Lachnospiraceae* over 4 weeks of supplementation (Figure 4A). In 2FL-supplemented *Il10*^-/-^ mice, the rise in *Lachnospiraceae* (from 10.6 ± 5.5% to 37.1 ± 12.1%) was matched by a pronounced decrease in *Bacteroidaceae* (from 51.1 ± 8.4% to 33.0 ± 7.8%). In 3FL-supplemented *Il10*^-/-^ mice, the increase of *Lachnospiraceae* was accompanied by a decrease in *Ruminococcaceae*, while *Bacteroidaceae* remained unchanged. Further OTU assignment revealed that the increment of *Lachnospiraceae* in 2FL-supplemented animals was almost exclusively due to expansion of the species *Ruminococcus gnavus* (from 4.0 ± 3.2% to 31.1 ± 8.3%). On the other hand, the main representants of *Bacteroidaceae* consisted of *Bacteroides acidifaciens* and *Bacteroides vulgatus*, which decreased during 2FL supplementation from 26.3 ± 5.5% to 16.0 ± 3.4% and from 12.6 ± 3.5% to 7.4 ± 2.8%, respectively (Figure 4B). In 3FL-supplemented animals, expansion of *R. gnavus* (from 2.1 ± 1.5% to 18.7 ± 5.2%) also mainly accounted for the increase in *Lachnospiraceae*, which, however, yielded in a lower total abundance of this microbe after D28. In clear contrast to 2FL treatment, 3FL supplementation showed a tendency to induce an increase of *B. vulgatus* from 7.8 ± 4.5% to 15.1 ± 1.8%. Altogether, these data indicate that oral supplementation of *Il10*^-/-^ mice with 2FL and 3FL led to increased abundance of the *Lachnospiraceae R. gnavus* especially upon ingestion of 2FL.

**FIGURE 5:**
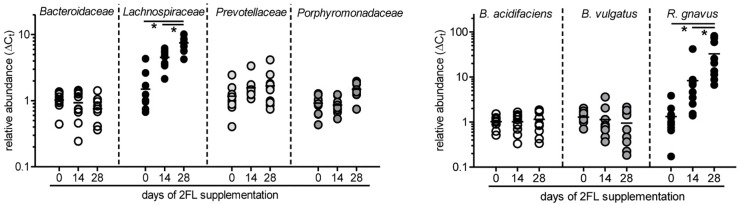
Relative expansion of bacterial taxa after 2FL supplementation. Bacterial DNA was isolated from fecal pellets of 2FL supplemented *Il10*^-/-^ mice on day 0, 14, and 28. Bacterial composition of each sample was assessed by qPCR analysis. Bacterial taxa at family level (left) and species level (right). To obtain relative taxa expansion, 16S DNA was used for total bacteria normalization and samples were referenced to mean taxa abundance on a D0 sample that represents the median according to ΔΔC_t_ method. Individual data points from 10 biological replicates of 3 independent supplementation experiments (*N* = 10). *P*-values were determined by one-way ANOVA with Dunnett Post-test and are indicated by asterisk (^∗^) if significant (*p* > 0.05).

To confirm the growth promoting effect of 2FL on *Lachnospiraceae* and *R. gnavus* in particular, microbiota composition was additionally analyzed by quantitative real-time PCR in mice that were supplemented for 4 weeks after weaning with Lac or 2FL. The relative abundance of *Bacteroidaceae*, *Lachnospiraceae*, *Prevotellaceae*, and *Porphyromonadaceae* in fecal samples was determined by using specific primers for these groups. In line with the data obtained from 16S rRNA sequencing, the increase of *Lachnospiraceae* could be clearly confirmed (Figure 5). At the species level, the concomitant increase of *R. gnavus* upon 2FL supplementation was also evident. The analysis of additional mice, however, did not confirm the decrease in *Bacteroidaceae* levels to a significance threshold.

### Growth Promoting Effect of Fucosylated Oligosaccharides on *R. gnavus*

**FIGURE 6:**
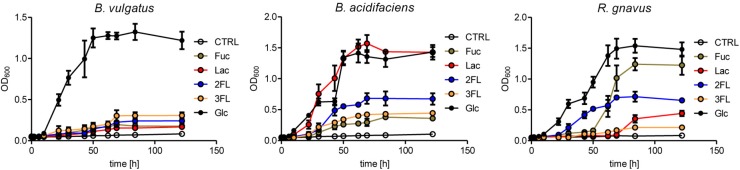
Mono- and oligosaccharide utilization by bacterial cultures. *B. acidifaciens*, *B. vulgatus*, and *R. gnavus* were cultured in chopped meat medium (CMM) to a final OD_600_ of 0.8. Then they were transferred to Hungate tubes with growth-limiting YCFA medium containing 20 mM of fucose (Fuc), glucose (Glc), 2-fucosyllactose (2FL), 3-fucosyllactose (3FL), or 10 mM of lactose (Lac) to an initial OD_600_ of 0.05. Negative control tubes (CTRL) contained YCFA medium without any carbohydrate substrate. Growth was monitored until stationary phase was reached. Mean values are shown from triplicates of 2 independent growth experiments. Error bars represent SD.

The expansion of *R. gnavus* during supplementation with fucosylated oligosaccharides likely reflected a growth advantage conferred by these carbohydrates on that bacterial species. *R. gnavus* has been previously shown to efficiently utilize mucin glycans including the oligosaccharides 2FL and 3FL as carbon source (Crost et al., 2013). We confirmed the growth of *R. gnavus* ATCC 29149 and compared it to the proliferation of *B. acidifaciens* JCM 10556 and *B. vulgatus* ATCC 29327 cultured in minimal medium supplemented with glucose, fucose, Lac, 2FL, and 3FL as unique carbohydrate sources. The three bacterial strains had comparable growth kinetics when glucose was provided as sole nutrient (Figure 6). Whereas *B. vulgatus* failed to efficiently utilize the other carbohydrates, the growth of *B. acidifaciens* was robust with glucose and Lac, and moderate with 2FL and 3FL, indicating that *B. acidifaciens* expressed α-fucosidase and β-galactosidase enzymes able to release galactose and glucose as nutrients. *B. acidifaciens*, however, only metabolized free fucose inefficiently. *R. gnavus* presented the opposite picture with a robust growth in presence of fucose (Figure 6). The onset of proliferation was delayed to 50 h post-seeding, indicating that growth was probably either conditional on the selection of mutants with fucose-metabolizing ability, or the expression of a fucose-utilization operon. 2FL, but not 3FL, initiated an early proliferation of *R. gnavus*, suggesting that the bacteria express a linkage-specific fucosidase conferring the preferential release of α1–2 linked fucose. This linkage specificity may be related to the stronger and earlier expansion of *R. gnavus* observed in 2FL- vs. 3FL-supplemented mice (Figure 4B).

**FIGURE 7:**
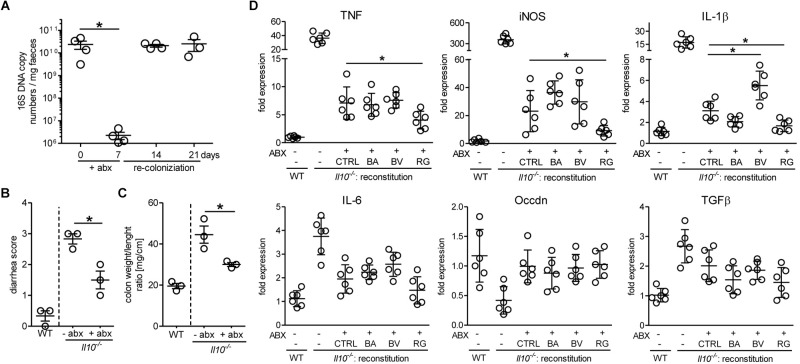
Antibiotic treatment and reconstitution of *Il10*^-/-^ mice. Mice were treated with antibiotics for 7 days and microbiota allowed to re-colonize for 14 days. **(A)** Efficacy of antibiotic treatment was assessed by analyzing 16S rRNA copy numbers in DNA isolated from fecal pellets from *Il10*^-/-^ mice by qPCR using the ΔΔC_t_ method. **(B) + (C)** Colon weight/length ratio **(B)** and diarrhea score **(C)** were analyzed to determine colitis severity. **(D)** Inflammatory marker gene expression after reconstitution of antibiotic-treated *Il10*^-/-^ mice with different bacterial strains or culture medium (CTRL). β-actin was used for normalization and the sample that represents median from untreated WT mice was used as reference sample according to the ΔΔC_t_ method. Biological replicates are shown from 1 **(A–C)** or 2 **(D)** independent experiments (*N* = 3–6). Error bars indicate SD. *P*-values were determined by one-way ANOVA with Dunnett Post-test and are indicated by asterisk (^∗^) if significant (*p* > 0.05).

Because the growth of pure bacterial strains *in vitro* does not reflect the competition for nutrients prevailing in the gastrointestinal tract, we have assessed the ability of *R. gnavus* to repopulate the gut microbiota of *Il10*^-/-^ mice after antibiotic treatment. In general, the induction of spontaneous colitis in *Il10*^-/-^ mice is strongly associated with the gut microbial community (Sellon et al., 1998). Treatment of mice with broad spectrum antibiotics for 7 days drastically reduced the levels of intestinal bacteria, which readily returned to initial levels a week after cessation of the treatment (Figure 7A). This antibiotic treatment decreased intestinal inflammation for at least 14 days before restoration of the microbiota as assessed by diarrhea score (Figure 7B) and colon weight/length ratios (Figure 7C). Antibiotic-treated *Il10*^-/-^ mice were reconstituted with 10^9^ CFU of either *B. acidifaciens*, *B. vulgatus*, or *R. gnavus* to determine the effect of these bacterial strains on inflammation. In case of *R. gnavus* and *B. acidifaciens*, reconstitution yielded a pronounced increase of these bacteria, which was higher than the initial abundancies of the respective strains in *Il10*^-/-^ mice. By contrast, *B. vulgatus* only expanded marginally after oral reconstitution (Supplementary Figure S3). The reconstitution of mice with *Bacteroides* did not decrease intestinal inflammation beyond the stage reached by natural recolonization after antibiotic treatment (Figure 7D). Reconstitution with *R. gnavus*, however, further alleviated inflammation as observed by the reduced expression of the inflammation markers iNOS, TNF and IL1β in distal colon tissue (Figure 7D). These results indicate that the expansion of *R. gnavus* mediated by 2FL supplementation contributed to decreased colitis in *Il10*^-/-^ mice.

### Differential Activation of Bone Marrow-Derived Dendritic Cells by Bacterial Strains

**FIGURE 8:**
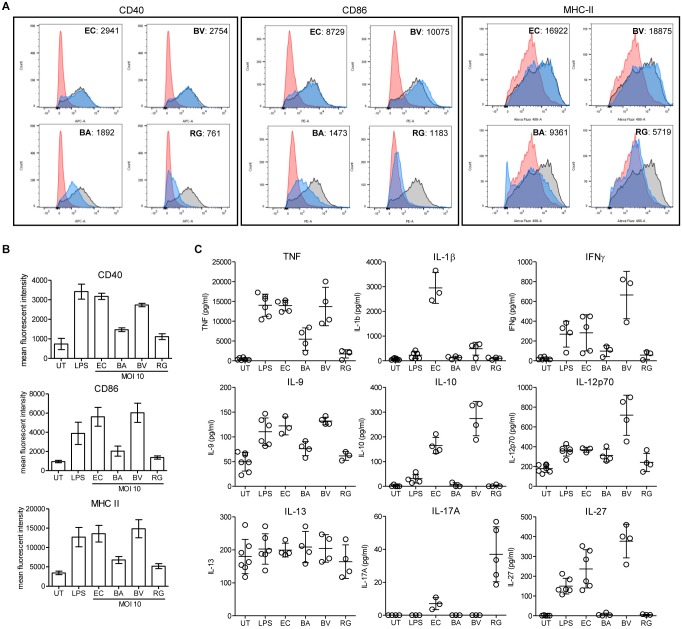
BMDC stimulation by fixed bacteria. Bone marrow cells were isolated from WT mice and *in vitro*-differentiated into BMDCs and stimulated with PBS (red histogram), 100 ng/ml LPS (black/gray histogram) or fixed cultures of *E. coli* (EC), *B. acidifaciens* (BA), *B. vulgatus* (BV), or *R. gnavus (*RG) at a MOI of 10 (blue histogram). Activation of BMDCs was determined by expression of activation markers or cytokine secretion. **(A)** Representative histograms of activation marker expression of CD40, CD86, and MHC-II. **(B)** Mean fluorescent intensity of activation markers in 3 independent experiments. **(C)** Cytokine secretion of stimulated BMDCs. Biological triplicates are shown (*N* = 3–5) from 3 independent experiments. Error bars indicate ± SD. *P*-values were determined by one-way ANOVA with Dunnett Post-test and are indicated by asterisk (^∗^) if significant (*p* > 0.05).

To evaluate the impact of *R. gnavus* on inflammation and immune reactions, we have compared the activation of dendritic cells mediated *R. gnavus*, *B. acidifaciens*, *B. vulgatus*, and *Escherichia coli*. Bone marrow-derived dendritic cells (BMDCs) from WT mice were incubated with fixed bacteria and cell activation was assessed by measuring the surface expression of the activation markers CD40, CD86, and MHC-II, and by analyzing cytokine profiles. Stimulation of BMDCs with *E. coli* and with pure lipopolysaccharide (LPS) strongly activated BMDC as evidenced by increased expression of the markers CD40, CD86, and MHC-II (Figure 8A). Stimulation with *B. vulgatus* also yielded a strong induction of activation markers, whereas *B. acidifaciens* and especially *R. gnavus* only yielded a moderate expression of CD40, CD86, and MHC-II (Figure 8B). The bacteria tested induced different cytokine responses in BMDCs. As expected, *E. coli* and LPS elicited a strong pro-inflammatory cytokine response characterized by elevated TNF, IL-1β, IFN-γ, IL-9, IL-12, and IL-27 production (Figure 8C). *B. vulgatus* also induced a potent pro-inflammatory cytokine response with elevated TNF, IFN-γ, IL-9, IL-12, and IL-27. The cytokine responses to *B. acidifaciens* and *R. gnavus* stimulation were more restricted and mitigated. Interestingly, *R. gnavus* induced a strong production of IL-17A, probably originating from T-cells present in the enriched BMDCs fractions. Although IL-17A production is increased in IBD (Fujino et al., 2003), the role of this cytokine in the etiology of the disease is still controversial. Neutralization of IL-17A was even shown to be ineffective at curing patients with moderate to severe Crohn’s disease (Hueber et al., 2012). Surprisingly, only the pro-inflammatory bacteria *E. coli* and *B. vulgatus* elicited the production of IL-10 (Figure 8C), which is essential in the maintenance of immune homeostasis in the gut. The low stimulatory effect of *R. gnavus* on BMDC is probably related to its surface molecules, which lacks LPS. The low stimulatory effect of *R. gnavus* on immune cells therefore confirms the beneficial impact on intestinal inflammation achieved through 2FL supplementation in *Il10*^-/-^ mice.

## Discussion

Whereas monosaccharides are mainly resorbed in the small intestine, oligosaccharides transit and remain intact down to the microbe-rich colon. Our study in *Il10*^-/-^ mice demonstrated that the oligosaccharide 2FL specifically promoted the expansion of the *Lachnospiraceae R. gnavus*, thereby decreasing the development of colitis in young animals after weaning. By this means, 2FL could have an onset-delaying effect in individuals with a colitis-prone predisposition even in the post-lactation period. This observed effect was definite for 2FL, while sialylated oligosaccharides such as 3SL or 6SL, and even the structurally similar 3FL failed to induce colitis protection, indicating that the mechanism involves a high linkage specificity. The colitis-reducing effect was accompanied by a shift in the microbiota that involved a significant increase of *Lachnospiraceae*. Interestingly, 2FL could also be shown to induce significant shifts of microbiota in a cohort of adult human individuals, showing that the impact of the bacterial community is not limited to early phase of life and opening new potential therapy options (Elison et al., 2016). A potential beneficial effect of 2FL was also seen in a different study using a dextran sulfate sodium model of colitis. However, the expansion of *Lachnospiraceae* was only transient and eventually overtaken by bacteria of the *Barnesiella* genus after 6 weeks (Weiss et al., 2014). Apart from IBD, 2FL supplementation has been shown to be beneficial in necrotizing enterocolitis models of newborn rats and pigs. However, this was not observed in combination with a change in microbiota and needs to be further evaluated with intervention on human newborns (Autran et al., 2016; Cilieborg et al., 2016).

Accumulating evidence suggests the involvement of certain bacterial taxa of the microbiota to pathological conditions, such as IBD (Zeng et al., 2017). Nevertheless, a substantial cause-impact relationship of specific bacterial taxa to disease phenotype is yet unidentified, probably due to the multifactorial pathological mechanism of IBD. However, bacteria in general are believed to be a key factor in IBD progression, whereas their role in the etiology of IBD is open to discussion. The results of our experiments with mice that were treated with antibiotics clearly show that microbiota plays a major role in the progression of colitis in the *Il10*^-/-^ model. In our study, the 2FL-mediated reduction of colitis in the *Il10*^-/-^ model was accompanied by the outgrowth of *R. gnavus*. In contrast to this finding, other studies and meta-analyses revealed an expansion of *R. gnavus* to be indicative for several pathological conditions, such as Crohn’s disease and allergic diseases in infants (Chua et al., 2018; Sokol et al., 2018). However, these findings need to be handled with caution because of their correlative nature. On the one hand, *R. gnavus* could just be a bystander as mucus-degrading bacteria, profiting from mucus changes in inflammatory conditions, such as increased mucus thickness and upregulation of Muc2 secretion, which have both been described for Crohn’s disease (Smirnova et al., 2001; Strugala et al., 2008; Crost et al., 2013). Intriguingly, *R. gnavus* abundance was significantly higher in *Il10*^-/-^ mice compared to WT animals at steady state as shown in the present study. On the other hand, taxonomic profiling of stool samples from IBD patients revealed that blooming of *R. gnavus* is accountable to only one specific clade that is particularly adapted to oxidative stress given by the inflammatory environment (Hall et al., 2017). Moreover, *Lachnospiraceae* and especially *R. gnavus* were found to be highly frequent in early-life gut colonization (Sagheddu et al., 2016). Interestingly, a study showed that in 2-months old breast-fed infants *Lachnospiraceae* consisted exclusively of *R. gnavus*, while a cow’s-milk based formula resulted in a more diverse *Lachnospiraceae* community (Tannock et al., 2013).

While a colitis-protective effect was only seen with 2FL, outgrowth of *R. gnavus* was observed in 2FL as well as 3FL supplemented animals. One mechanism possibly explaining the anti-inflammatory effect of 2FL against 3FL may be the faster rate of expansion of *R. gnavus* and the higher relative abundance of this bacterium achieved with 2FL. Supplementation of 2FL might enable *R. gnavus* to contribute to a microbial environment that restricts the colonization with bacteria triggering robust inflammatory responses. By contrast, the slower expansion of *R. gnavus* mediated by 3FL was less efficient at dampening the expansion of other bacterial taxa to the same extent. The SCFA propionate has been shown to be protective for colonocytes by alleviating intestinal inflammation through promotion of regulatory T cell development (Arpaia et al., 2013; Smith et al., 2013). The increased proprionate production observed in 2FL-treated mice (see Figure 3) may thus contribute to the overall protective effects of the oligosaccharide treatment. Submucosal invasion of bacteria at local sites of epithelial disruption, which is frequently seen in the *Il10*^-/-^ model, leads to activation of immune cells, release of pro-inflammatory cytokines, such as TNF, IL-6 and IFN-γ and ultimately even to more epithelial cell damage and increased leukocyte infiltration (Gunther et al., 2015). We could show that *Bacteroidaceae* strains, and especially *B. vulgatus*, are much more potent in the activation of dendritic cells and secretion of those cytokines when compared to *R. gnavus*. This might be a possible mode of action how 2FL supplementation and ensuing *R. gnavus* expansion leads to a reduction of *Bacteroidaceae* in the microbial community and therefore to a dampened inflammation response. A drastic reduction of *Bacteroidaceae* might also be the mechanism providing protection against colitis in antibiotic-treated *Il10*^-/-^ mice. However, our study failed to provide any direct evidence for this mechanism given that the reduction of *Bacteroides* species such as *B. vulgatus* did not reach a significant level. Alternatively, the relative proportion of *Bacteroidaceae* measured by 16S rRNA sequencing may not represent changes in absolute amounts, but indirectly reflect the relative outgrowth of other bacterial taxa. Apart from that, we cannot exclude that 2FL also exerts a direct microbiota-independent effect on intestinal epithelial or submucosal immune cells. Some oligosaccharides have been shown to alter epithelial and immune cell responses, leading to differential gene expression that might influence TLR-mediated activation or tissue healing (Kuntz et al., 2009; He et al., 2014). However, at least for DCs recent studies have shown that 2FL lead to no direct modulation of differentiation or maturation by 2FL (Perdijk et al., 2018). A potential indirect mechanism for differential immune cell polarization might include 2FL-mediated *R. gnavus* outgrowth that resulted in a selective expression of the cytokine IL-17 in this study. Taken together, our data suggest that supplementation in the post-lactation period with the prebiotic 2FL induces a beneficial microbial community shift in terms of colitis. Hence, it might be an interesting and promising supplement for diet in IBD patients in terms of disease progression.

## Materials and Methods

### Mouse Models

All animal experiments conducted in this study were performed in compliance with the Swiss Animal Protection Ordinance and approved by the Veterinary Office of the Canton of Zurich, Switzerland. B6.129P2-*Il10^tm1Cgn^*/J mice, termed *Il10*^-/-^ throughout this article, were obtained from Jackson Laboratories and bred on a C57BL/6J background (WT). All mice were held in light-cycled and climate-controlled housing conditions in an IVC facility in T2L cages, received laboratory chow diet (KLIBA extrudat #3436, Provimi Kliba, SA, Switzerland) and sterilized drinking water *ad libitum*, if not stated otherwise. For experimental conditions, 3–4 mice were held in a cage. At the end of the experiment mice were euthanized using CO_2_. Animals were not reused for multiple experiments. Biological material from the same mouse was subjected to different types of analyses.

### Animal Experiments

*Il10*^-/-^ mice were weaned 21 days after birth and supplemented with a 5 mM solution of either 2-fucosyllactose (2FL), 3-fucosyllactose (3FL), 3-sialyllactose (3SL), 6-sialyllactose (6SL) (Glycom A/S, Hørsholm, Denmark) or D-lactose (Sigma-Aldrich, Buchs, Switzerland) in sterile filtrated water *ad libitum* for 28 days. For depletion of intestinal microbiota, 4–6 weeks old mice were supplied with 0.5 g/L vancomycin (AppliChem), 1 g/L ampicillin (VWR), 1 g/L neomycin (Fisher Bioreagents) and 0.2% aspartame (Sigma) in drinking water for 6 days. Once per day, mice received intragastric gavage of 250 μL of a 1 g/L metronidazole (Sigma) solution. After antibiotic treatment, mice were treated with 10% PEG 3000 solution (Sigma) in drinking water to flush the remaining bacteria and antibiotic. Neo-colonization with specific bacterial strains was done by intragastric gavage of 250 μL containing 10^9^ CFU of the respective strain in PBS. Fecal pellets were collected to monitor changes of the microbiota.

### Bacterial Cultures

Anaerobic cultures of *Ruminococcus gnavus* [ATCC 29149, (Moore et al., 1976)], *Bacteroides acidifaciens* [JCM 10556, (Miyamoto and Itoh, 2000)] and *Bacteroides vulgatus* [ATCC 29327, (Eggerth and Gagnon, 1933)] were expanded in anoxic chopped meat medium (DSMZ, #78 including hemin and vitamin K_1_) in Hungate tubes at 37°C. To determine single carbon source substrate utilization, cultures were transferred to Hungate tubes containing YCFA medium (Duncan et al., 2002) supplemented with the respective carbon source as indicated and cultured at 37°C. Growth was monitored twice a day for at least 5 days by determination of OD_600_. The *E. coli* EHV2 strain that was isolated and characterized previously, was cultured in LB medium at 37°C under aerobic conditions (Huang et al., 2015).

### Diarrhea Score

The most remarkable changes in individual consistency of feces are represented in the diarrhea score, assessed in a blinded manner and modified after Sakai et al. (2013). 0: normal, dry and solid feces; 1: swollen, moist feces; 2: soft and mucous feces; 3: wet and shapeless feces; 4: bloody diarrhea.

### *Trans*-Epithelial Integrity

Mice were intragastrically gavaged with 200 μL of a 60 mg/ml FITC-Dextran (MW 3000-5000; Sigma) solution in ddH_2_O 90 min prior to euthanasia. Blood serum was collected after centrifugation at 1500 ×*g* for 15 min. Serum fluorescence intensity was measured using a multi-detection microplate reader (Tecan Infinite^®^ M200 Pro, Switzerland) at an excitation wavelength of 485 nm and an emission wavelength of 535 nm. FITC concentration was calculated from a standard curve using serial dilutions of FITC–dextran.

### Histology and Colitis Score

A tissue sample of 2 cm from distal colon was isolated, briefly washed with PBS, fixed in 10% neutral buffered formalin (Sigma) for at least 24 h and processed for paraffin embedding and sectioning. Histopathological analysis to determine colitis scores was performed on deparaffinized 5 μm Hematoxylin and Eosin (Sigma) stained tissue sections. Sections were scored individually by an independent investigator blinded to the type of treatment as described previously (Steidler et al., 2000).

### RNA Isolation and qPCR

Tissue from proximal or distal colon was homogenized with a Precellys^®^ 24 tissue homogenizer (Bertin Instruments) and RNA was isolated using Trizol reagent (Sigma) after manufacturer’s instructions. Total RNA (1 μg) was reverse transcribed using High-Capacity cDNA Reverse transcription Kit (Applied BioSystems^TM^) following manufacturer’s instructions. For quantitative PCR reactions KAPA SYBR^®^ Fast (Sigma) Master mix was used and amplified with a CFX95 Touch^TM^ Real-Time System with each 50 nM of the following oligonucleotides: iNOS for: 5′-AGC CTT GCA TCC TCA TTG GG-3′; iNOS rev: 5′-CCT TTG AGC CCT TTG TGC TG-3′; TNF for: 5′-GGC CTC CCT CTC ATC AGT TC-3′; TNF rev: 5′-CAC TTG GTG GTT TGC TAC GAC-3′; IL-1b for: 5′-GCT GGA GAG TGT GGA TCC CAA G-3′; IL-1b rev: 5′-TGC TGA TGT ACC AGT TGG GG-3′; IL-6 for: 5′-CAC GGC CTT CCC TAC TTC AC-3′; IL-6 rev: 5′-GCC ATT GCA CAA CTC TTT TCT C-3′; TGFβ for: 5′-TGG AGC AAC ATG TGG AAC TC-3′; TGFβ rev: 5′-GTC AGC CGG TTA CCA-3′; Occdn for: 5′-CCC TGA CCA CTA TGA AAC AG-3′; Occdn rev: 5′-TTG ATC TGA AGT GAT AGG TG-3′. Cycling conditions were 50 cycles at 95°C for 10 s and 60°C for 30 s after an initial denaturation at 95°C for 3 min. All targets included an exon-exon junction-spanning oligonucleotide to reduce genomic DNA amplification. ΔΔc_t_ method was applied to analyze the relative gene expression with β-actin as an endogenous normalization reference and a sample representing the median from lactose treated WT mice used as control sample.

### Isolation, Amplification and Sequencing of Fecal Pellet Bacterial DNA

Bacterial DNA was isolated from fecal pellets using the QIAamp DNA stool Mini Kit (QIAGEN) in compliance to manufacturer’s instructions. 20 ng of bacterial DNA were used to amplify the V3-V4 16S rRNA region, using oligonucleotides with Illumina adapters sequences: V3F 340-356: 5′-CTT TCC CTA CAC GAC GCT CTT CCG ATC TCC TAC GGR AGG CAG CAG-3′ and V4R 805-786: 5′-GGA GTT CAG ACG TGT GCT CTT CCG ATC TGG ACT ACH VGG GTW TCT AAT-3′. Amplicon sequencing was performed using an Illumina-MiSeq^®^ system (Genotoul, France). The bioinformatics pipeline Quantitative Insight Into Microbial Ecology (QIIME) open source software package (Versions 1.8.0 and 1.9.0) was used for taxonomic classification of 16S rRNA gene sequencing (Caporaso et al., 2010). Raw datasets were processed by merging, trimming, chimeric reads purging and OTU construction following the UPARSE pipeline (Edgar, 2013). Assignment of taxonomic units was done using the Greengenes database (Version 13.8) as a reference database (DeSantis et al., 2006).

For determination of bacterial taxa by qPCR 50 nM of specific oligonucleotides were mixed with KAPA SYBR Fast (Sigma) Master mix and amplified with a CFX95 Touch^TM^ Real-Time System. Cycling conditions were 50 cycles at 95°C for 10 s and 60°C for 30 s after an initial denaturation at 95°C for 3 min. The following primers were used: total bacteria 16S for (Eub338F): 5′- ACT CCT ACG GGA GGC AGC AG-3′; rev (Eub518R): 5′- ATT ACC GCG GCT GCT GG -3′ (Guo et al., 2008); *R. gnavus* for: 5′-CCA ATT ACG GAA AGC TGG AT-3′; rev: 5′-TCT GCT TTC CAT GTA TCT TCA CA-3′ (Crost et al., 2013); *B. acidifaciens* Rec A for: 5′-AAC CTG ATA ACG GTG AGC AGG CG-3′; rev: 5′-GCT GAC AGC CGA GGT CAA TTT G-3′; *B. vulgatus* for: -5′-GGA GGG GAA AGA CTT ATT TTG C-3′; rev: 5′-TTC CAC CAC TTC TGC CGA C-3′ (Huang et al., 2015). *Lachnospiraceae* for: 5′-CGG TAC CTG ACT AAG AAG C-3′; rev: 5′-AGT TTY ATT CTT GCG AAC G-3′ (Rinttila et al., 2004); *Bacteroidaceae* for: 5′-AAG GTC CCC CAC ATT GG-3′; rev: 5′-GAG CCG CAA ACT TTC ACA A-3′ (Hermann-Bank et al., 2013); *Porphyromonadaceae* for: 5′-ACG CGC GAA TCC CGA AAA CC-3′; rev: 5′- CTT CGT ACG CTC CTT GCG GTT G-3′ (Ramirez-Farias et al., 2009). The lack of significant sequence similarity of the selected primers with unrelated bacterial sequences was confirmed by BLAST analysis. ΔΔc_t_ method was applied to analyze the relative taxon abundance with universal bacterial 16S rRNA (Eub338F and Eub518R) as an endogenous normalization reference and a sample representing the median of samples from day 0 was used as a control sample.

### Quantitative Analysis of Cecal SCFA

Mouse cecal contents were isolated and centrifuged at 4°C and 8,000 × g for 10 min. SCFA analysis was done by HPLC using a cation-H refill cartridge (30 × 4.6 mm) and an Aminex HPX-87H column at a flow rate of 0.4 ml.min^-1^ for 60 min and eluted with 10 mM sulphuric acid solution. Quantification was done by detection of refractive index. The concentration was calculated by integral area comparison with authentic standard solutions.

### *In vitro* Differentiation and Stimulation of BMDCs

Bone marrow cells were isolated from tibia, femur and ilium of C57BL/6 WT mice as previously published by Matheu et al. (2008) with minor modifications. Briefly, red blood cell lysis was performed by incubation with ammonium-chloride-potassium lysis buffer (150 mM NH_4_Cl, 10 mM KHCO_3_, 0.1 mM EDTA) for 5 min on ice. Isolated bone marrow cells were cultured and differentiated in Iscove’s Modified Dulbecco’s Medium (Gibco), with 10% FCS, GlutaMAX^®^ supplement (Gibco), antibiotic-antimycotic solution (Gibco), and 1 mM sodium pyruvate (Gibco). Cells were differentiated using 30 ng/mL GM-CSF (Peprotech) and 40 ng/mL IL-4 (Peprotech), replated after 6 days and stimulated after 11 days of isolation. For stimulation, *E. coli* EHV2, *B. acidifaciens*, *B. vulgatus*, and *R. gnavus* were fixed in 2% paraformaldehyde for 15 min at RT, washed with sterile PBS and reconstituted to a density of 5 × 10^8^ CFU/mL. BMDCs were stimulated with either LPS (O111:B4; 100 ng/mL) or the respective fixed bacterial strains with a multiplicity of infection (MOI) of 10. After 18 h, cells were washed with PBS and harvested by scraping. Activation of BMDCs was determined by fluorescence-labeled antibodies (Biolegend): FITC α-mouse MHC-II (clone M5/114.15.2; #107696); APC α-mouse CD40 (clone 3/23; #124612); PE α-mouse CD86 (clone GL-1; #105008). Samples were acquired using a FACSCanto^TM^ II flow cytometer (BD Biosciences).

### BMDC Cytokine Secretion Analysis

Expression and secretion of cytokines after BMDC stimulation was determined by ProcartaPlex^TM^ Multiplex immunoassay (Thermo Fisher Scientific) according to manufacturer’s instructions. Snap-frozen supernatant from BMDCs was diluted 1:2 and incubated with bead mixes B (Lot#140144000) and C (Lot#144194000). Absolute cytokine concentrations were assessed by a serial dilution of Standard Mix A (Lot#146651101).

### Statistical Analysis

All multiple group comparisons were performed using one-way ANOVA with Dunnett *post hoc* test to compare all groups to a control indicated in the respective figure legend. Continuous variables are shown as ± SD and a *p*-value < 0.05 was considered significant. Throughout this study, all data points are shown, without excluding any outliers. All statistical analyses were performed using GraphPad Prism (Version 5.03).

## Data Availability

The raw sequence data has been submitted to the European Nucleotide Archive (ENA) database and can be found under the project number PRJEB31580.

## Ethics Statement

This study was carried out in accordance with the recommendations of the Swiss Animal Protection Ordinance. The protocol was approved by the Veterinary Office of the Canton of Zürich, Switzerland.

## Author Contributions

TH designed and conceived the study and secured the funding. TG performed and analyzed the experiments and prepared the figures. JGG performed the BMDC FACS experiments. MH performed the histopathological scoring. AG and CL analyzed the 16S sequencing data. TG and TH wrote the manuscript.

## Conflict of Interest Statement

The authors declare that the research was conducted in the absence of any commercial or financial relationships that could be construed as a potential conflict of interest.
