# Supplementary material for: Alleviation of Intestinal Inflammation by Oral Supplementation With 2-Fucosyllactose in Mice
Source: Front Microbiol. 2019 Jun 19;10:1385. doi: 10.3389/fmicb.2019.01385 (PMC6593069; doi:10.3389/fmicb.2019.01385)
Supplement: Supplementary file 1 [file Data_Sheet_1.docx]

Supplementary Material

# Supplementary figures

**A**

**B**

**Supplementary Fig. 1:** Inflammatory marker gene expression in proximal colonic tissue of IL10-/- **(A)** and distal **(B)** colonic tissue of WT mice. Expression levels of inflammation markers were assessed by qPCR. Expression of the iNOS, the cytokines IL-1β, IL-6, TGFβ, TNF as well as the tight junction protein Occludin was determined. β-actin was used for normalization and one sample from a Lac supplemented WT mouse was used as reference sample according to the ΔΔCt method. Each group consisted of at least six animals (N = 6-8), error bars indicate SD.

**A**

**B**

**C**

**Supplementary Fig. 2: (A)** Alpha diversity of 16S sequencing from fecal DNA from day 28 of supplementation with Lac, 2FL, 3FL, 3SL and 6SL samples shown by Chao1 index. Gray lines indicate individual samples, red line indicates mean ± SD (N = 27). (**B)** Comparison of bacterial family abundance in WT vs. *Il10^-/-^* samples before supplementation experiments (D0). Individual samples are shown and mean is indicated (N = 15). (**C)** Individual differences in WT vs. *Il10^-/-^* mice of OTUs at family level before supplementation (D0). Each group consisted of at 15 animals (N = 15), error bars indicate SD. P values were determined by student’s T test and are indicated by asterisk (*) if significant (p > 0.05).

**Supplementary Fig. 3: *In vivo* expansion of bacterial strains during reconstitution after abx treatment in *Il10^-/-^* mice.** Relative abundancy of bacteria was determined by qPCR with normalization to total bacterial DNA and samples were referenced to mean taxa abundance on D0 according to ΔΔCt method. Individual data points from 6 biological replicates of 2 independent experiments (N = 6).
